# Supplementary material for: Perinatal outcome of meconium stained amniotic fluid among labouring mothers at teaching referral hospital in urban Ethiopia
Source: PLoS One. 2020 Nov 13;15(11):e0242025. doi: 10.1371/journal.pone.0242025 (PMC7665744; doi:10.1371/journal.pone.0242025)
Supplement: Attachment — Submitted filename: Reviewer.docx [file pone.0242025.s002.docx]

July 10, 2020

To: PLOS ONE Editor in chief.

Dear Editor in chief.

We would like to thank the editor and reviewers for their thoughtful review of the manuscript. They raise important issues and their inputs are very helpful for improving the manuscript. We agree with almost all their comments and we have revised our manuscript accordingly. We respond below in detail to each of the editor comments. We hope that you find our responses satisfactory and that the manuscript is now acceptable for publication

Looking forward hearing from you soon

Sincerely,

Lemi B Tolu (MD, Assistant prof of obstetrics and gynecology).

Saint Paul’s Millennium Medical College (SPHMMC)

Department of Obstetrics and Gynecology

Addis Ababa, Ethiopia.

Email: lemi.belay@gmail.com

**Dear editor**

Thanks for thoughtful review of the manuscript. Below is point by point response to raised concerns and how we changed the manuscript according to the comments.

**#Editor**

1. Please ensure that your manuscript meets PLOS ONE's style requirements, including those for file naming.

Response: Dear editor thank you very much manuscript revised according to the journal requirement.

2. We noticed you have some minor occurrence of overlapping text with the following previous publication(s), which needs to be addressed:

Response: Thank you a lot, concern addressed (line 42-62)

**Reviewer #1:**

A well thought study looking at the perinatal outcome of meconium stained amniotic fluid (MSAF) among laboring mothers at teaching hospital in Ethiopia. This study brings out important information regarding the outcomes of MSAF in Ethiopia.

**Response**: Thank you very much for energizing comment, below is the line by line response to concerns.

#Corrections:

1. Line 42 –Can be corrected as: Meconium stained amniotic fluid is usually seen in 12 to 16% of deliveries.

Response: Corrected (Line 42)

2. Line 47- Can be corrected as: Though its controversial to differentiate physiologic or pathologic meconium staining of amniotic fluid, there are few shreds of evidence that indicates its association (instead of associated) with increased meconium aspiration syndrome, respiratory distress, neonatal sepsis and perinatal mortality.

Response: Corrected (line47)

3. Line 77: Gestational age was (instead of is) calculated from reliable last normal menstrual period or early ultrasound done before 24 weeks and those with an unknown date or without early ultrasound were (where) excluded.

Response: Corrected (line 85)

4. Line 168: All thin (grade one) stained fluid gave birth vaginally, while 80(86%) of thick (grade two and three) group underwent (instead of undergone) operative delivery.

Response: Corrected (Line 178)

5. Line 190: Our study also highlighted that 86% of (thich) thick stained 190 amniotic fluid group (grade two and three) undergoes operative delivery which indicates the risk of fetal heart rate abnormality with meconium staining

Response: Corrected (Line 199)

6. Line 202: Several investigators

Response: Corrected (Line 212)

7. Line 209: In the current research, MAS was (has) diagnosed in 6.3 % a baby of the stained fluid group, which is 2.3 times compared to a non-stained fluid group
8. Table :2 P value is not in line for prim gravida and Induced labor.

**Response:** corrected (table 2)

#Suggestions

1.It is unclear what the primary objective of this study was in the introduction. To determine the outcomes for physiologic v pathologic meconium staining or to compare MSAF outcomes with clear amniotic fluid. My recommendation is to have a clear objective in your introduction.

**Response:** Thank you, suggestion addressed (68-69)

2.My suggestion is to compare Meconium aspiration syndrome between different types of MSAF and not with clear amniotic fluid and to analyze the outcome differences between different types of MSAF

**Response:** Dear reviewer thank you very much, the wrong comparison with clear amniotic fluid is corrected as Meconium aspiration syndrome was seen in 9(6.3%) stained fluid group (line 183-184). Unfortunately, among those 9 (6.3%) who developed MAS we didn’t collected the subgroups on thickness of amniotic fluid.

3. Were there any other conflicting variables like scheduled repeat C- section? Were those mothers excluded

**Response**: Dear reviewer our population are those in labour, admitted to labour ward.

4. My suggestion is also to compare the outcomes based on different Gestational age (Early term, term and post term).

**Response**: Dear reviewer we did gestational age matched data collection to control possibility of gestational age confounding the outcomes.

**Reviewer #2:**

#General notes: The text should be proofread for spelling, spacing and grammatical mistakes. The overall readability is borderline, but in several spots, it is very difficult to understand what the authors mean. Please have the text proofread and edited. I appreciate the authors efforts to have the text proofread by a professional service, but it seems the editing service used by authors was substandard.

**Response**: Dear reviewer, thank you very much. We reviewed the whole manuscript to address the spelling, spacing and grammatical mistakes concern.

#Specific comments:

1. Line 68 and 69 do not belong to methods since you report the results. Please move it to the result section.

**Response**: Concern addressed (line 76-77 and line 144-145)

2. Line 158. You define prolonged rupture of membranes as longer than 12h. It seems to be different from 18 hours used in the US. Can you explain somewhere the choice of this interval?

**Response**: Dear reviewer yes, it is true different definitions is used for prolonged rupture of membrane. Pediatrics commonly use 18 hrs. in our hospital to consider for risky of neonatal sepsis. In Obstetrics 12 hrs. is commonly used

3. Lines 172-174. MSAF is one of the requirements to establish MAS diagnosis (your lines 105-106). How the patients with no MSAF were diagnosed with MAS?

**Response:** Thank you the wrong comparison corrected as Meconium aspiration syndrome was seen in 9(6.3%) stained fluid group (line 183-184)

4. In the description of demography (Table 1), the religion part might be misunderstood by some readers. Please specify if the Orthodox religion means Orthodox Judaism or Orthodox Christianity. I would spell out religions as Christian with subgroups of Eastern Orthodox and Protestant. Alternatively, I would spell out Orthodox Judaism and Christian (Protestant).

**Response**: Thanks, concern addressed as Orthodox Christian (Table 1)

5. Table 2. Please specify if the diabetes was type one or pregnancy-induced.

**Response**: Thanks, concern addressed as pregestational DM (Table 2)

It is interesting, that the follow-up was performed via a cell phone connection. Please, comment if the population of the woman included in the study or admitted to the hospital truly represent the population of Addis-Ababa. I would expect that only small proportion of the population could afford cell phones. Please clarify if I am wrong.

**Response**: Dear reviewer currently most Ethiopians, even rural one had cell phone. The fact that our study is in Addis Ababa, capital city makes the follow up easy as our study population were from Addis Ababa or surrounding city.
