# Supplementary material for: Perinatal outcome of meconium stained amniotic fluid among labouring mothers at teaching referral hospital in urban Ethiopia
Source: PLoS One. 2020 Nov 13;15(11):e0242025. doi: 10.1371/journal.pone.0242025 (PMC7665744; doi:10.1371/journal.pone.0242025)
Supplement: Attachment — Submitted filename: Response to reviewers .docx [file pone.0242025.s003.docx]

August 27, 2020

To: PLOS ONE Editor in chief.

Dear Editor in chief.

We would like to thank the editor and reviewers for their thoughtful review of the manuscript. They raise important issues and their inputs are very helpful for improving the manuscript. We agree with almost all their comments and we have revised our manuscript accordingly. We respond below in detail to each of the editor comments. We hope that you find our responses satisfactory and that the manuscript is now acceptable for publication

Looking forward hearing from you soon

Sincerely,

Lemi B Tolu (MD, Assistant prof of obstetrics and gynecology).

Saint Paul’s Millennium Medical College (SPHMMC)

Department of Obstetrics and Gynecology

Addis Ababa, Ethiopia.

Email: lemi.belay@gmail.com

**Dear editor**

Thanks for thoughtful review of the manuscript. Below is point by point response to raised concerns by reviewer#2.

Reviewer #2: Dear Authors,

1. I would like to stress it again, I like the idea of your paper and this data should be published in the best possible form.

**Response**: dear reviewer thanks a lot. We will also do our best to extent of our potential and consult people in our network.

2. I am still not satisfied with the English part. I see the other reviewer helped you with some of that, but the text still needs a major proofreading. For example, your home city is misspelled on the first page. It is spelled as “Addis ababa” . The title has words started with capital letters in random order. Choose one style. Capitalize them all or none. Obviously, the first word in the sentence and the name of the country should be capitalized, should you chose not capitalizing all the words. And so on and so on for the entire paper including papers.

**Response:** Dear Reviewer thanks for the comment. Title edited accordingly and the whole text proofreading done by two authors (me and Garumma Tolu Feyissa). However, “Addis Ababa” is normally spelled like that though we might find “Addis Abeba” in some texts.

3. Your result part of the summary is too descriptive. The readers will be looking for specific numbers of incidence of MAS in MSAF, Apgar scores, NICU admission rates and so on. Your description what was higher or lower is not enough. Please, provide the data of the incidence of MSAF and MAS in the summary. Please add the proportion of primigravida mothers in your population to the summary, if the space allows. You could cut out a big proportion of the statistics part form the summary to include more data.

**Response**: Dear reviewer the abstract section was edited accordingly (lines 31-40)

4. It is interesting, that most of your patient population were primigravida. Perhaps you may explain this in discussion, why your group is so different from the rest of the country with average fertility rate over 4. Also, your group is very different form the rest of the country in regard of the literacy. You report that 6-8% of your mothers were illiterate, while in general illiteracy rate in Ethiopia is reported to be more than 50%. Perhaps you would like to mention this and explain why your group is different. Could it be explained by better education in Addis Ababa or just the population your hospital serves? My primary concern is that you group is not true representative of population of the country in terms of education, wealth and accessibility to health care. This in turn could result in different accessibility and desire to use the health care services. With all value of your data it is important to mention the limitations.

**Response**: Dear reviewer thank you very much for the input, the concerns where addressed (lines 192-193 and lines 225-232).

5. Perhaps I have missed it, but I was not able to see the reason for Cesarean section delivery in your patients. It is important to indicate whether it was scheduled, or the obstetricians had to do it emergently for any reason. Perhaps you also could discuss if the Cesarean section delivery places a baby at risk of MAS or the Cesarean section serves as a rescue for infants who are about to develop MAS.

**Response**: Included with indications for both groups (lines 171-175 and lines 198-199)

6. Table 4 – I do not see a reason for removal of the MAS incidence from this table. Isn’t it the main outcome?

**Response**: Dear reviewer MAS was removed from table because of the contradiction of MAS among clear amniotic fluid group and expressed in sentences (lines 178-179) as “Meconium aspiration syndrome was seen in 9(6.3%), stained fluid group”
