# Supplementary material for: Perinatal outcome of meconium stained amniotic fluid among labouring mothers at teaching referral hospital in urban Ethiopia
Source: PLoS One. 2020 Nov 13;15(11):e0242025. doi: 10.1371/journal.pone.0242025 (PMC7665744; doi:10.1371/journal.pone.0242025)
Supplement: Attachment — Submitted filename: Editor respons.docx [file pone.0242025.s004.docx]

September 2020, 2020

To: PLOS ONE Editor in chief.

Dear Editor in chief.

We would like to thank the editor and reviewers for their thoughtful review of the manuscript. They raise important issues and their inputs are very helpful for improving the manuscript. We agree that the manuscript requires editing for English grammar and usage. Hence, the manuscript was copy edited by two authors (one research fellow at Drexel university/USA) and Pre-Publication Support Service (PRESS). PRESS is Michigan University/USA based publication support organization providing manuscript preparation and copy editing. We hope that you find our revision satisfactory and that the manuscript is now acceptable for publication

Looking forward hearing from you soon

Sincerely,

Lemi B Tolu (MD, Assistant prof of obstetrics and gynecology).

Saint Paul’s Millennium Medical College (SPHMMC)

Department of Obstetrics and Gynecology

Addis Ababa, Ethiopia.

Email: lemi.belay@gmail.com
