# Supplementary material for: Perinatal outcome of meconium stained amniotic fluid among labouring mothers at teaching referral hospital in urban Ethiopia
Source: PLoS One. 2020 Nov 13;15(11):e0242025. doi: 10.1371/journal.pone.0242025 (PMC7665744; doi:10.1371/journal.pone.0242025)
Supplement: Attachment — Submitted filename: Editor respons.docx [file pone.0242025.s005.docx]

September 25, 2020

To: PLOS ONE Editor in chief.

Dear Editor in chief.

We would like to thank the editor for the thoughtful review of the manuscript. They raise important issues and their inputs are very helpful for improving the manuscript. We agree with all points raised and modified the manuscript accordingly. We hope that you find our revision satisfactory and that the manuscript is now acceptable for publication

Looking forward hearing from you soon

Sincerely,

Lemi B Tolu (MD, Assistant prof of obstetrics and gynecology).

Saint Paul’s Millennium Medical College (SPHMMC)

Department of Obstetrics and Gynecology

Addis Ababa, Ethiopia.

Email: [lemi.belay@gmail.com](mailto:lemi.belay@gmail.com)

Dear Editor

Thank you very much. Please see the point by point response to raised concerns

1. In Results section: Line 156: Either add women or patients after pregnant.

**Response**: Done line 136

Line 156: Add Patients after postpartum

**Response**: Done line 137

2. Line 157: add and after interview.

**Response**: Done line 137

3. Discussion:236: you have typo, I think you mean to say non-stained

**Response**: thank you very much, yes, it is to mean non-stained, corrected line 212

4. 238: take out similar, its redundant.

**Response**: Done, line 214

5. 239: Please rephrase the line, it’s not clear. Do you mean to state " 6.3% of babies in stained fluid group"

Response: Done, line 215 to 216

6. Conclusion: 260: I would advise making line more generalized than giving specific timeline, the way its worded is a strong statement to make. I am assuming you are concluding it based on limited data you mentioned in line 234 and 235 in discussion. Please consider changing it to " Knowing the high risk of early neonatal death we advise early postnatal follow up should be considered for infants born to mothers with thick MSAF."

**Response**: Dear editor thank you very much for such beautiful thought and input, it means a lot. Corrected as “knowing the high risk of early neonatal death we advise early postnatal follow up should be considered for infants born to mothers with thick MSAF”, line 236-237
